# Beyond Virulence: A Multi-Trait Development Framework for Advancing Entomopathogenic Fungi into Mycoinsecticides

**DOI:** 10.3390/microorganisms14102275

**Published:** 2026-10-06

**Authors:** Min Luo, Jian Wu, Hongyu Zhang, Nan Liao, Wei Chen, Qiongbo Hu

**Affiliations:** 1 Ganzhou Green Leafy Vegetables Technology Innovation Center, Ganzhou Polytechnic, Ganzhou 341000, China; 2 State Key Laboratory of Green Pesticides, College of Plant Protection, South China Agricultural University, Guangzhou 510642, China

**Keywords:** mycoinsecticides, green pesticide, multi-trait evaluation, production fitness, formulation, field performance

## Abstract

Entomopathogenic fungi (EPF) are promising microbial control agents, but high laboratory virulence alone does not ensure successful development into reliable mycoinsecticides. This review proposes a sequential multi-trait framework organized around three stages: biological candidate qualification, product qualification, and translational qualification. Biological qualification evaluates target-specific pathogenicity, host–pathogen ecological fit, abiotic stress tolerance, and trait stability. Product qualification assesses whether a candidate can be manufactured reproducibly as viable and infective propagules, formulated, dried, stored, and delivered without unacceptable loss of performance. Translational qualification tests whether the formulated product provides consistent pest suppression under relevant greenhouse, semi-field, and field conditions and fits the intended deployment scenario. The framework distinguishes intrinsic strain traits from process-shaped product traits and treats the strain–propagule–process combination as the relevant product-stage unit. It also separates development-limiting weaknesses from limitations that may be mitigated through production, formulation, or deployment technologies, supporting decisions to advance, redirect, or discontinue candidates. This framework provides a practical basis for linking laboratory screening with manufacturability and field performance and for identifying traits that merit prospective validation as predictors of successful mycoinsecticide development.

## 1. Introduction

**Figure 1:**
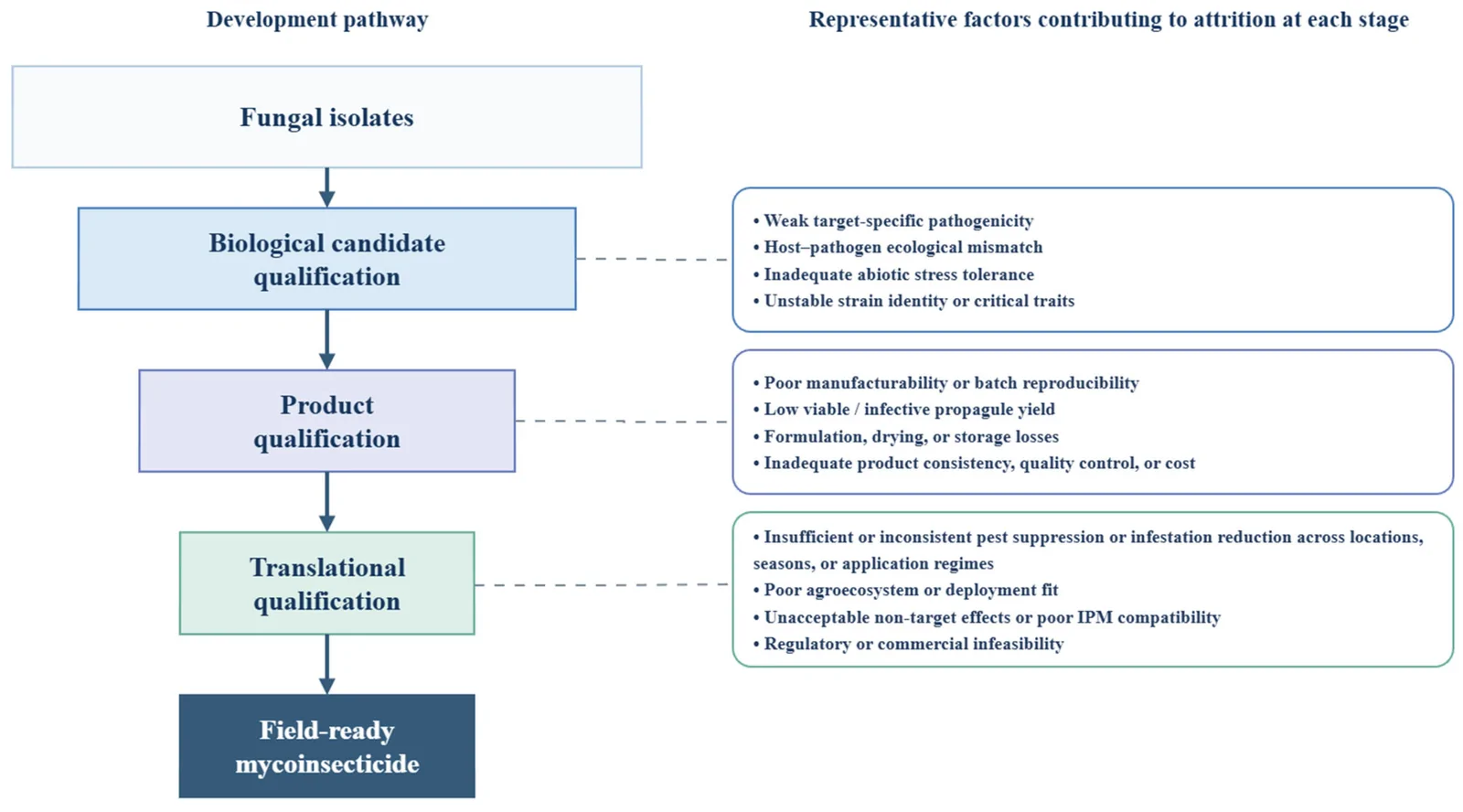
Progressive attrition across the three qualification stages involved in advancing entomopathogenic fungi toward mycoinsecticide development. The factors shown are representative rather than exhaustive and may vary with the target pest, production system, formulation, deployment scenario, and regulatory context.

Entomopathogenic fungi (EPF), particularly species of *Beauveria*, *Metarhizium*, and *Cordyceps*, are widely studied microbial control agents for arthropod pests in agricultural, forestry, veterinary, and public health systems. Their infective propagules can adhere to and penetrate the arthropod cuticle without requiring ingestion, broadening their potential against pests with diverse feeding habits and facilitating their integration into integrated pest management (IPM) programs [1]. Current regulatory records show that EPF-based bioinsecticides are registered across several major agricultural markets, including Brazil, the European Union, and the United States [2]. Nevertheless, many isolates that exhibit high pathogenicity in laboratory assays fail to progress into robust and consistently effective mycoinsecticides. This gap reflects attrition across biological candidate qualification, product qualification, and translational qualification, each of which introduces distinct constraints on fungal performance (Figure 1) [3].

The conventional starting point for EPF development is virulence screening against a target pest. Mortality, median lethal concentration (LC_50_), median lethal time (LT_50_), infection rate, and mycosis are commonly used to identify promising isolates [4]. These endpoints are essential because adequate pathogenic activity is a prerequisite for further development, but their predictive value depends heavily on assay conditions. Host species and developmental stage, inoculum dose, exposure route, temperature, relative humidity, and observation period can all influence the apparent ranking of isolates. This limitation becomes evident when laboratory results are compared with field performance. *Simplicillium lanosoniveum* caused 72.85–95.20% mortality across developmental stages of *Aleurodicus rugioperculatus* at the highest laboratory concentration, whereas preliminary field applications reduced egg and nymph populations by only 57.8% and 56.3%, respectively [5]. Field efficacy can also become difficult to interpret when pest pressure is intrinsically low. In a field study using honey bees to disseminate *Beauveria bassiana* against *Lygus lineolaris* in canola, low pest population densities prevented the collection of sufficient insects to assess mortality reliably in open-field plots [6]. Field performance and its interpretation can also be influenced by temperature, humidity, solar radiation, microclimate, and other site-specific conditions [7].

Accordingly, candidate evaluation has expanded beyond pathogenicity to include abiotic stress tolerance. Tolerance to temperature, UV radiation, moisture limitation, and other habitat-specific stresses strongly influences whether infective propagules remain viable and active long enough to initiate infection [8]. Production introduces additional constraints related to yield, fermentation efficiency, scalability, batch consistency, and physiological quality, while drying, formulation, and storage further affect viability, persistence, and retained bioactivity [9]. Many of these traits are also process-dependent. Production media may be solid or liquid, and their nutritional composition can substantially influence both propagule phenotype and fungal biochemical output. Carbon and nitrogen availability are relevant to both solid-state and submerged cultivation: solid substrates such as cereals differ in nutrient composition and C:N ratio and can alter conidial yield, hydrophobicity, thermotolerance, and virulence, whereas carbon and nitrogen sources in liquid media can affect blastospore production, stress tolerance, virulence, and shelf stability. Culture conditions can also modify extracellular enzyme activity and secondary-metabolite production. For example, destruxin production in *Metarhizium anisopliae* is influenced by nutrient availability and culture conditions, particularly nitrogen availability, although in vitro metabolite production does not necessarily correlate directly with insecticidal activity [10,11,12,13]. Development potential cannot be predicted reliably from virulence alone.

Recent studies have increasingly adopted multi-trait approaches to improve candidate selection. Idali et al. evaluated virulence, conidial production, and thermotolerance across 49 pathogenic isolates and proposed an integrated phenotypic screening pipeline for *Beauveria bassiana* and *Metarhizium robertsii* [14]. Production studies likewise show that the highest-yielding treatment does not necessarily provide the best combination of virulence and stress tolerance, supporting the joint evaluation of productivity and biological quality [15]. However, most multi-trait studies still focus on a single stage of development. After initial screening, production conditions, propagule type, harvesting, drying, formulation, and storage can reshape the phenotype expressed at application [16]. These observations support a useful distinction between strain phenotype, process-shaped product phenotype, and field performance, with field performance emerging from interactions among the product, target pest, environment, and delivery strategy.

This framework lies in integrating biological candidate qualification, product qualification, and translational qualification into a single decision-oriented development pathway. It further distinguishes strain phenotype from process-shaped product phenotype, treats the strain–propagule–process combination as the relevant product-stage unit, and separates development-limiting weaknesses from those that may be technologically compensated (Table 1). Together, these elements provide a basis for deciding whether a candidate should advance, be redirected for optimization, or be discontinued.

This review is an integrative narrative review. Relevant studies were identified through targeted searches of Web of Science, Google Scholar, and PubMed, supplemented by reference list screening of key studies and reviews. Search terms covered entomopathogenic fungi, pathogenicity, host–pathogen ecological fit, abiotic stress tolerance, production and propagule characteristics, formulation and storage, and greenhouse and field performance. Studies were included when they provided evidence relevant to one or more stages of the proposed development framework, with priority given to peer-reviewed primary studies and recent reviews. Because the included studies varied substantially in fungal species, target pests, propagule types, experimental conditions, and application systems, the evidence was synthesized qualitatively rather than quantitatively. The literature search was updated through August 2026. Throughout this review, evidence that a trait influences fungal biology or product performance is distinguished from evidence that the trait has been validated as a predictor of field efficacy; the latter remains considerably more limited and context-dependent.

## 2. Beyond Virulence: Qualification of Entomopathogenic Fungal Candidates

### 2.1. Target Pathogenicity and Host–Pathogen Ecological Fit

Virulence remains the first requirement for advancing an EPF isolate. Mortality, LC_50_, LT_50_, infection rate, and mycosis provide practical measures of pathogenic activity and allow rapid comparison among large numbers of isolates [17]. However, these endpoints describe performance against a particular host under a defined set of assay conditions. The same isolate may perform differently as the target species, developmental stage, dose, exposure route, or observation period changes. Inoculum age and storage history are particularly relevant because prolonged storage or culture maintenance can alter germination, physiological vigor, and retained pathogenicity [18]. For *S. lanosoniveum*, LC_50_ increased from 4.72 × 10^4^ conidia/mL against eggs of *A. rugioperculatus* to 5.92 × 10^5^ conidia/mL against fourth instar nymphs/pupae, demonstrating substantial stage-dependent susceptibility [5]. Stage-dependent susceptibility has also been demonstrated for *Beauveria bassiana*. In a study with *Phauda flammans*, conidia of *B. bassiana* PfBb stored at 4 °C for 360 days caused significantly lower mortality in fifth- and sixth-instar larvae than in first- to fourth-instar larvae under 25 ± 1 °C, 75 ± 5% relative humidity, and a 13L:11D photoperiod [19].

In many laboratory bioassays, conidial density is routinely manipulated to standardize inoculum dose. Pathogenicity, however, is not determined by inoculum concentration alone. Successful infection involves a sequence of biological and biochemical processes, including adhesion and germination, cuticle penetration mediated by extracellular enzymes such as proteases, chitinases, lipases, proliferation within the host, and the production of secondary metabolites that can disrupt host physiology or immune responses. These biochemical traits can help explain differences in virulence among fungal isolates, but they should complement rather than replace whole-insect bioassays because the production of individual metabolites in vitro does not necessarily correlate directly with mortality or speed of kill [13,20].

Host identity is equally important. EPF range from relatively broad host range generalists to more specialized pathogens, and high performance against one host does not guarantee comparable efficacy against another. For example, *Metarhizium rileyi*, a lepidopteran specialist, was more virulent against *Spodoptera frugiperda* than the more generalist *M. anisopliae* [21]. Even within a single pest species, plant-mediated effects can alter susceptibility; host plants may influence insect physiology, cuticular chemistry, or fungal development and thereby change mortality and subsequent sporulation [22].

Candidate evaluation should reflect the intended biological and operational context. For a direct-contact foliar product, rapid infection of exposed stages may be especially important, whereas fungi intended for autodissemination or population-level transmission requires additional attention to conidial transfer, mycosis, and sporulation on cadavers. Isolate-dependent differences in horizontal transmission and cadaver sporulation have been demonstrated in *Beauveria* and *Metarhizium* tested against banana weevils [23]. Candidate qualification should consider both pathogenic activity and host–pathogen ecological fit. The latter includes compatibility with the intended pest species and life stage, exposure route, host behavior, and target habitat. High pathogenicity under a single assay condition does not necessarily indicate a good ecological match for the intended infection scenario.

### 2.2. Abiotic Stress Tolerance

After target-specific pathogenicity and host–pathogen ecological fit have been established, candidate qualification should assess tolerance to the abiotic stresses expected during infection. Temperature, solar radiation, humidity, water availability, and related abiotic stresses directly affect conidial germination, growth, survival, and infectivity [24]. Solar radiation should be considered in terms of both spectral composition and light regime. UV-B is generally among the most damaging components for exposed fungal propagules, but UV-A and visible light can also influence germination, sporulation, stress tolerance, and pathogenicity, and responses under light and dark conditions may differ among isolates. Rossouw et al. compared no-light, visible-light, and UV (280–400 nm) exposure and found isolate-dependent differences in survival and pathogenicity among *Beauveria* and *Metarhizium* isolates [25]. In comparative UV-B assays, some *B. bassiana* isolates lost culturability rapidly, whereas others and several *Metarhizium* isolates retained greater activity after prolonged exposure [26]. Abiotic screening should consider the wavelength composition and light–dark regime relevant to the intended application environment rather than UV tolerance alone.

Temperature illustrates why abiotic stress tolerance should also be evaluated in relation to infection rather than fungal growth alone. Thermal performance curves for *M. brunneum* and *M. flavoviride* showed that growth optima and virulence responses were not always aligned; *M. brunneum* maintained a broader optimal temperature range for virulence, whereas *M. flavoviride* displayed a narrower and more complex thermal response [27]. More broadly, thermal performance varies among *Metarhizium* isolates and is partly associated with geographic origin, while fungal growth under a given temperature regime does not necessarily predict virulence in the host [28]. Abiotic screening should include biologically relevant endpoints such as germination and infection across the expected temperature range rather than relying solely on radial growth.

Geographic origin may provide useful context for potential climatic adaptation, but local origin should not be treated as a substitute for experimental evaluation of tolerance within the intended temperature, humidity, and radiation regime [29]. Abiotic screening at this stage should determine whether the candidate can retain germination, growth, and infective capacity within the temperature, humidity, water availability, and radiation conditions expected during the infection process.

### 2.3. Trait Stability and Candidate Advancement

A favorable phenotype is useful only if it can be reproduced. EPF traits can change during repeated subculture, host passage, or prolonged maintenance, affecting virulence, sporulation, germination, and other characteristics relevant to biological control [30]. Serial culture on artificial media has repeatedly been associated with attenuation in some fungal isolates, while passage through a susceptible host can restore or enhance virulence. For two *M. guizhouense* isolates, repeated insect passage restored or increased virulence, whereas ten serial subcultures reduced virulence in one isolate but had little effect on the other [31]. This isolate-specific response indicates that stability cannot be assumed from species identity or generalized from a single strain.

Passage effects are also context-dependent. Repeated passage of *B. bassiana* through melon, tomato, and cotton improved aspects of endophytic colonization while maintaining stable insect virulence [22]. In contrast, another study found that insect and plant passaged *M. robertsii* reisolates showed reduced virulence relative to the parental strain, whereas repeated culture on Sabouraud medium produced only minor changes [32]. These contrasting results show that passage does not produce a universal directional effect. Candidate qualification should evaluate whether key phenotypic and, where relevant, biochemical traits remain within an acceptable range after the propagation or maintenance procedures expected during development.

During product development, stability testing should be incorporated at defined checkpoints rather than assessed only during initial isolate screening [33]. Strain identity should be verified from authenticated stock cultures and rechecked after repeated culture, production cycles, or scale-up using appropriate molecular identification methods and, where available, strain-specific markers [34]. Phenotypic consistency should be monitored by reassessing critical traits such as propagule viability, including germination where appropriate, sporulation, growth, target-specific pathogenicity, propagule yield, and relevant stress tolerances [35]. Where specific extracellular enzymes or bioactive secondary metabolites are known to contribute substantially to the pathogenicity or product performance of a candidate, their activity or production profile should also be monitored during repeated culture, production, or scale-up. Passage number, inoculum age, storage history, and production conditions should also be recorded so that changes can be traced to specific stages of propagation or manufacture [36]. When substantial drift in identity or critical performance traits is detected, production should be restarted from an authenticated reference stock rather than continued through additional serial passage. Where broad-spectrum use is intended, candidate qualification should also examine pathogenicity across multiple target pest species or populations [37]. Extensive multi-location validation, however, is more informative after production and formulation because field performance depends on the final product as well as local pest populations, crop conditions, climate, and application practices [38]. Where exposure of beneficial arthropods is foreseeable, preliminary non-target screening should assess pathogenicity, infection, mortality, or other relevant adverse effects in representative pollinators, predators, or parasitoids under relevant exposure conditions [39]. Such early screening can identify candidates with unacceptable intrinsic hazards, whereas broader compatibility assessment is more informative after formulation, dose, application route, and deployment conditions have been defined.

These considerations define the first decision gate in mycoinsecticide development. A candidate should advance when it combines adequate target-specific pathogenicity, host–pathogen ecological fit, sufficient abiotic stress tolerance within the intended use window, and trait stability. Some deficiencies are development-limiting at this stage, including consistently weak pathogenicity against the intended pest or life stage, failure to germinate or infect within the target temperature range, or severe instability of critical traits. Other weaknesses may be potentially compensable. Moderate sensitivity to UV radiation or desiccation, for example, may justify continued development if the underlying pathogenicity and ecological fit are strong because later production or formulation strategies can sometimes improve abiotic stress tolerance [40]. The purpose of Gate 1 is to eliminate candidates with fundamental biological limitations while retaining those whose remaining weaknesses may be addressed during product development. This distinction prevents early screening from demanding an unrealistically perfect isolate and provides a clear transition from candidate qualification to the technological questions addressed in product qualification.

## 3. From Candidate to Product: Production, Propagule, and Formulation Fitness

### 3.1. Production Fitness and Scale-Up

After biological qualification, an EPF candidate must demonstrate manufacturability: it should be produced reproducibly at practical scale while retaining biological quality and remaining compatible with downstream processing and storage. Production fitness encompasses batch-to-batch consistency, production time, yield of viable and infective propagules, substrate and process requirements, scalability, downstream recovery and stabilization, storage performance, and cost. Solid-state fermentation remains widely used for aerial conidia, whereas submerged fermentation can generate blastospores, submerged conidia, microsclerotia, and other propagules under more controlled conditions [41]. Production requirements are often specific to species and strain, so high laboratory virulence does not guarantee manufacturability.

Yield alone is an insufficient measure of production fitness because culture conditions can modify propagule quality. In *B. bassiana*, cereal substrates affected conidial yield, hydrophobicity, thermotolerance, and virulence; rice favored abundance and virulence, whereas wheat produced more hydrophobic and thermotolerant conidia [10]. Likewise, the nutritional treatment producing the highest yield and enzymatic activity in *Metarhizium rileyi* did not produce the most virulent conidia; another treatment increased virulence by approximately 35% [15]. Production optimization should evaluate productivity and biological quality together.

Cost-effective and sustainable production media can improve commercial feasibility, provided that propagule quality is maintained. Production media may include organic nutrient sources and inorganic mineral supplements, and their suitability should not be judged by material class or purchase price alone. Cereal substrates such as rice and wheat are widely used for solid-state production of aerial conidia, whereas submerged fermentation can use lower-cost agro-industrial materials such as cottonseed flour, corn bran, corn gluten meal, inactive yeast, or autolyzed yeast [11]. Selection should consider nutrient composition and C:N ratio, batch consistency, local availability, renewability or potential for by-product valorization, requirements for pretreatment and sterilization, propagule yield and quality, compatibility with scale-up, and subsequent harvesting, drying, and formulation. Production media should be selected according to the yield of viable, biologically competent propagules per unit time and cost together with resource efficiency and downstream processing requirements, rather than propagule number alone [42].

Manufacturability should be evaluated across the complete production chain rather than at the fermentation step alone. A high-yielding process has limited value if propagule viability, infectivity, or stress tolerance varies markedly among batches, if harvesting or drying causes substantial losses, or if the resulting material cannot be stabilized and stored economically. Reproducible production of biologically competent propagules, together with compatibility with downstream processing and storage, provides a stronger indication of production fitness than total propagule yields alone. These process-dependent changes also reinforce the need to evaluate the strain together with the propagule type and production process rather than treating strain identity as the sole development unit.

### 3.2. Propagule Type and Process-Shaped Product Phenotype

Propagule type is a major determinant of product performance. Aerial conidia are widely used because of their infectivity and relative tolerance to desiccation, whereas submerged fermentation can produce blastospores, submerged conidia, microsclerotia, and other structures with different physiological properties. The most appropriate propagule depends on the intended production process, formulation, and deployment environment [43].

Blastospores can be produced rapidly at high densities but are often more sensitive to drying and storage than aerial conidia. Their quality also depends strongly on fermentation conditions. In *B. bassiana*, changing the nitrogen source altered blastospore yield, thermotolerance, UV-B tolerance, virulence, and storage stability within the same strain. Microsclerotia provide a different strategy: these desiccation-tolerant structures can survive storage and subsequently produce infective conidia, making them attractive for soil applications [44]. Their performance after application, however, remains strongly affected by soil temperature, moisture, texture, and resident microbial communities. Soil microorganisms may restrict fungal establishment through competition for nutrients and ecological niches or through soil fungistasis, whereas rhizosphere-associated conditions can favor persistence and root colonization [45]. Conversely, the effects of introduced EPF on native soil microbial communities also require consideration, although available evidence suggests that these effects can be limited and are highly context-dependent.

Other propagules further illustrate that product phenotype cannot be inferred from strain identity alone. Microcycle conidia differ from aerial conidia in hydrophobicity, heat tolerance, and UV stability [46], while liquid culture cell aggregates of *B. pseudobassiana* retained germination after prolonged refrigerated storage and produced infective conidia after rehydration [47].

These observations support a distinction between strain phenotype and process-shaped product phenotype. The phenotype measured during isolate screening represents the strain under defined conditions, whereas the material eventually applied in the field has already been shaped by production, propagule differentiation, harvesting, and stabilization [48]. At this stage, the relevant development unit is better represented as a strain–propagule–process combination. Candidate ranking should be reassessed after production rather than assumed to remain unchanged from initial screening.

### 3.3. Formulation, Drying, and Storage Fitness

Produced propagules must survive formulation, stabilization, storage, transport, and application while retaining sufficient biological activity. The appropriate formulation depends on propagule properties and the intended delivery system. Dry formulations such as wettable powders, water-dispersible granules, and tablets facilitate storage, transport, dose standardization, and subsequent dispersion or application, whereas oil-based formulations can improve spreading and adhesion and may protect conidia from heat, desiccation, and other environmental stresses. Granular formulations are particularly suited to soil delivery because they allow localized placement and subsequent production of infective propagules, while encapsulation in biopolymer matrices can reduce physical and environmental stress and improve shelf life, handling, and controlled delivery [2]. Thus, formulation type affects not only biological protection but also product stability, transportability, application method, and field deployment.

Formulation fitness should be evaluated in relation to the intended deployment scenario rather than as an isolated product property. The same formulation may perform differently depending on the application route, target location, delivery system, and environmental conditions encountered after application [49]. Formulation selection should accordingly consider not only propagule protection and storage stability but also handling, deposition, adhesion, release, rainfastness, and compatibility with the intended application method and other components of the IPM program.

Carrier selection is an additional determinant of formulation fitness. Organic and biodegradable matrices, including cereal-based substrates and biopolymers such as alginate, starch, cellulose, and chitosan, may provide physical protection, nutrients, or controlled release, whereas mineral carriers such as diatomaceous earth, clay, and silica can improve handling and stabilization. Carrier chemistry can substantially affect propagule viability, storage stability, resporulation, and field persistence. For example, Metarhizium conidia retained substantially greater viability in several oil carriers than in diatomaceous earth during storage. Carrier selection should consider biological compatibility, biodegradability, physicochemical stability, cost, and potential environmental impacts in addition to formulation performance [2].

The capacity of formulation to mitigate environmental sensitivity has direct implications for candidate selection. Moderate UV or desiccation sensitivity may be acceptable when a practical formulation can provide sufficient protection [50]. However, formulation cannot compensate for every biological limitation, and compatibility must be tested for the selected propagule. Blastospores and other vegetative cells, for instance, may be more sensitive than aerial conidia to some surfactants and additives.

Drying is another critical transition because tolerance to dehydration differs substantially among propagule types. There is no universal drying temperature or final moisture content suitable for all EPF products; these parameters must be optimized for the strain, propagule, formulation, and drying method. Aerial conidia are generally more desiccation tolerant than vegetative blastospores, whereas resistant structures such as microsclerotia can withstand substantial dehydration and subsequently produce infective conidia after rehydration [51]. Nevertheless, even desiccation-tolerant propagules can be affected by drying rate, temperature, residual moisture, and storage conditions. In the B. bassiana IP 361 example described above, blastospores formulated with diatomaceous earth were air-dried for 20–24 h to a final moisture content of ≤6% before storage at 4 °C. Production conditions can also affect subsequent shelf life. In a multi-trait study of *B. bassiana* IP 361, six complex organic nitrogen sources were initially compared for blastospore production, stress tolerance, and virulence, after which cottonseed flour and autolyzed yeast were selected for scale-up and storage evaluation. Harvested blastospores were mixed with diatomaceous earth as an inorganic carrier, air-dried, and stored at 4 °C for up to 180 days. Shelf life was evaluated through viability and retained insecticidal activity during storage. Blastospores produced with cottonseed flour maintained more than 98% viability, whereas those produced with autolyzed yeast declined to approximately 89.5% after 180 days [11]. Shelf stability reflects interactions among strain, propagule, production history, formulation, and storage conditions. Storage assessment should extend beyond germination alone. Germination and CFU measurements can provide different estimates of viability, and residual insecticidal activity may persist even when in vitro indicators decline. Packaging, moisture, oxygen exposure, and temperature also influence long-term stability, especially under warm storage conditions.

When dehydration or thermal instability remains development-limiting, additional physiological and formulation-based strategies can be considered. Production conditions can modify endogenous protective solutes and subsequent stress tolerance; nutritional composition can influence intracellular mannitol and trehalose levels and the desiccation or heat tolerance of fungal propagules. Protective excipients can provide further stabilization during downstream processing. Skim milk combined with ascorbic acid has been used to protect spray-dried *B. bassiana* blastospores against osmotic and oxidative damage, whereas fructose, skim milk, and bentonite have been used to improve the survival of *M. robertsii* blastospores during spray drying [52]. Low-moisture packaging combined with oxygen and moisture scavengers can further prolong storage stability. Micro- and nanoencapsulation or coating approaches may provide additional physical protection against dehydration, heat, and radiation, but their effects on propagule release, biological activity, manufacturing cost, and scalability must be evaluated for each product.

A candidate should progress only when it can be produced reproducibly, converted into an appropriate propagule, formulated and stabilized with acceptable losses, and stored for a practical period while retaining biological activity. At this point, development has shifted from evaluating a promising isolate to evaluating whether a strain–propagule–process combination can function as a stable product [53].

## 4. From Product to Field: Translational Validation and an Integrated Development Framework

### 4.1. Greenhouse, Semi-Field, and Field Performance

A mycoinsecticide must ultimately demonstrate that laboratory activity translates into reliable pest suppression under realistic conditions. Greenhouse and semi-field trials should primarily be treated as proof-of-concept stages, where formulation, dose, application method, target-stage, and preliminary efficacy can be evaluated under partially controlled conditions [54]. Where exposure can be quantified reliably, concentration- or dose–response and time-to-mortality metrics such as LC_50_ and LT_50_ may also support comparative efficacy assessment and dose selection, although these metrics are generally most informative under standardized bioassay conditions [55]. These trials are useful for identifying promising deployment strategies but do not by themselves establish reliable field performance. Field validation should involve replicated testing under the range of environments and operational conditions relevant to the intended use, including, where appropriate, multiple locations and seasons [56]. Field success should be evaluated using endpoints relevant to the intended pest management objective rather than mortality alone. These may include pest suppression, infection and persistence, crop damage, yield or yield protection, application interval and treatment frequency, and the extent to which the product can be integrated with other components of the IPM program.

Laboratory performance may decline substantially after environmental exposure. *Metarhizium brunneum* F52 microsclerotia caused 85–100% mortality of annual bluegrass weevil adults under favorable laboratory conditions yet provided negligible greenhouse control and no significant larval suppression in field trials [57]. Low temperature, rainfall, microbial competition, and reduced insect–conidium contact were identified as likely constraints [58].

In contrast, *B. bassiana* successfully reduced *Plutella xylostella* populations in field trials, although efficacy depended on insect stage, formulation, application rate, and treatment frequency [59]. Commercial *B. bassiana* formulations also achieved approximately 85–87% reduction of *Thrips tabaci* in two-year onion trials [60].

There is no universal number of field trials that defines sufficient validation. The required evidence should reflect the intended use pattern and the environmental and biological variability the product is expected to encounter. Field validation should include replicated testing across relevant locations and seasons and, where appropriate, across pest populations or species, developmental stages, application rates, intervals, and treatment frequencies. Before commercialization or broad deployment, the evidence base should demonstrate reproducible performance across representative locations and seasons and where the intended use encompasses multiple target pest species or populations, across those targets. A single successful field trial provides evidence of feasibility but is insufficient by itself to establish the robustness required for commercialization [61,62].

### 4.2. Agroecosystem and Deployment Fit

Agroecosystem and deployment fit concerns whether the formulated product can function under the crop, soil, weather, management, and application conditions of its intended use. Unlike abiotic stress tolerance assessed during biological candidate qualification, performance at this stage emerges from interactions among the formulated product, target pest, crop, habitat, resident biological community, and delivery strategy [63]. Formulation and deployment should be evaluated as coupled components of product performance. A formulation that is suitable for foliar spraying may not be optimal for soil delivery, autodissemination, or other application systems, and its performance may change with rainfall, temperature, humidity, target location, and the use of compatible adjuvants or other IPM inputs [64].

Environmental compatibility may also extend to interactions with pre-existing agrochemical residues and inorganic contaminants. Residual pesticides may alter fungal germination, vegetative growth, sporulation, infectivity, or persistence, although their effects are strongly dependent on the active ingredient, formulation, residue concentration, fungal strain, and surrounding environmental conditions. Entomopathogenic fungi possess enzymatic systems capable of transforming several xenobiotics, and some Metarhizium species have been implicated in pesticide degradation. *M. robertsii* can demethylate methylmercury and reduce mercury accumulation in plants grown in contaminated soil, while *B. bassiana* has shown substantial biosorption capacity for several heavy metals [65,66]. These findings suggest potential environmental co-benefits of selected EPF strains, although most evidence derives from laboratory or specifically contaminated systems. Field validation should consider residual synthetic insecticides and other agrochemical residues, together with relevant inorganic contaminants, where these are likely to occur in the intended deployment environment [67].

Foliar applications are strongly affected by UV radiation, desiccation, rainfall, and temperature, whereas soil applications depend more heavily on soil moisture, pH, texture, organic matter content, nutrient status, temperature, microbial competition, and target location [63]. Rainfall after foliar application can remove deposited conidia from plant surfaces, although compatible sticker–spreader adjuvants and oil-based formulations can improve conidial deposition, adhesion, and rainfastness. Their effectiveness remains formulation-dependent and should be evaluated together with fungal viability and infectivity under realistic rainfall conditions. Application practices can also influence product quality immediately before use. Oil formulations, for example, can reduce heat-induced loss of conidial viability during tank mixing. Field conditions can also be manipulated operationally by selecting application windows that reduce environmental stress. Foliar applications may be scheduled during periods of lower solar radiation and more favorable humidity and temperature rather than during peak daytime exposure. In a two-year onion field study, *B. bassiana* and *M. anisopliae* applied in the evening to reduce adverse UV exposure and provide more favorable humidity and temperature conditions for fungal establishment. Application timing should be treated as part of deployment optimization rather than as a fixed operational detail [60].

Before deployment, packaging should maintain product quality during storage and distribution by limiting detrimental exposure to moisture, oxygen, temperature fluctuations, or other storage stresses. Delivery strategy should be matched to propagule biology and target pest developmental stage, behavior, and habitat. Granular formulations of *M. robertsii* microsclerotia and blastospores persisted under semi-natural pasture conditions, with fungal establishment and root colonization still detectable after 336 days [68]. Horizontal transmission and autodissemination systems provide another approach, particularly when direct spray coverage is difficult. In a two-year bitter gourd field study, an entomo-vectored horizontal transmission device combining butanone acetate with *B. bassiana* contaminated a mean 70.5% of *Bactrocera cucurbitae* adults and produced 81.2% mortality, together with reduced fruit infestation and increased marketable yield [69]. Horizontal transfer has also been demonstrated in the greenhouse whitefly *Trialeurodes vaporariorum*, where *M. anisopliae* contaminated adult donors transferred conidia to untreated recipient insects, causing mortality and mycosis in both groups, although recipient insects generally died more slowly [70]. These examples illustrate why conidial acquisition and retention, host contact, transfer efficiency, sporulation, and persistence can become as important as rapid mortality in transmission-based delivery systems.

Translational evaluation should explicitly include non-target arthropods and beneficial organisms because naturally occurring predators and parasitoids may already contribute substantially to pest suppression at the application site. Mycoinsecticide application should be evaluated within the existing biological control community rather than as an isolated intervention. Information from preliminary non-target screening should inform whether a candidate proceeds to formulation and should guide subsequent formulation, dose, application route, timing, and exposure-mitigation strategies. The compatibility of EPF with beneficial arthropods, including honeybees and other pollinators, predators, and parasitoids, may depend on fungal strain, formulation, dose, application route, timing, and exposure pathway. Potential effects may include direct infection or sublethal effects on beneficial arthropods, as well as indirect effects resulting from changes in prey or host availability. Root inoculation of strawberries with *M. robertsii* or *B. bassiana* reduced *Tetranychus urticae* populations across four commercial fields and two growing seasons without detectable effects on naturally occurring predatory mites [71]. Field application of a *Cordyceps fumosorosea* wettable powder against Metisa plana likewise caused no sustained adverse effect on the oil palm pollinator *Elaeidobius kamerunicus* [72]. Interactions with parasitoids can also vary among fungal–natural enemy combinations, emphasizing the need for direct compatibility testing [73]. Field validation should assess the natural enemy community before application and monitor target pest suppression together with the abundance, survival, infection, and functional activity of key predators, parasitoids, honeybees and other pollinators, and other relevant non-target arthropods after treatment. Where necessary, application timing, route, or spatial placement should be adjusted to reduce unnecessary exposure while preserving the contribution of existing natural enemies to pest suppression.

### 4.3. An Integrated Framework for Mycoinsecticide Development

The evidence reviewed above supports a sequential development framework in which different traits become decisive at different points along the isolate-to-product pathway. Early evaluation should focus on whether a fungal isolate possesses sufficient target-specific pathogenicity, is appropriately matched to the intended host and infection context, tolerates the relevant abiotic stress window, and maintains key traits with acceptable stability [74]. Candidates with consistently weak pathogenicity, severe host–pathogen mismatch, inadequate abiotic stress tolerance, or unstable critical traits are unlikely to justify further development [75].

Once a biologically promising candidate has been identified, evaluation should shift toward its capacity to become a reproducible product. At this stage, the relevant unit is better represented as a strain–propagule–process combination, because production conditions, propagule type, drying, formulation, and storage can substantially alter biological performance [2]. Product qualification should determine whether the candidate can be produced at practical scale, stabilized with acceptable losses, and stored for a realistic period while retaining sufficient activity. Formulation and deployment should also be treated as coupled design decisions, because formulation suitability depends on the intended application method, environmental conditions, target location, and compatibility with the surrounding IPM program.

**Figure 2:**
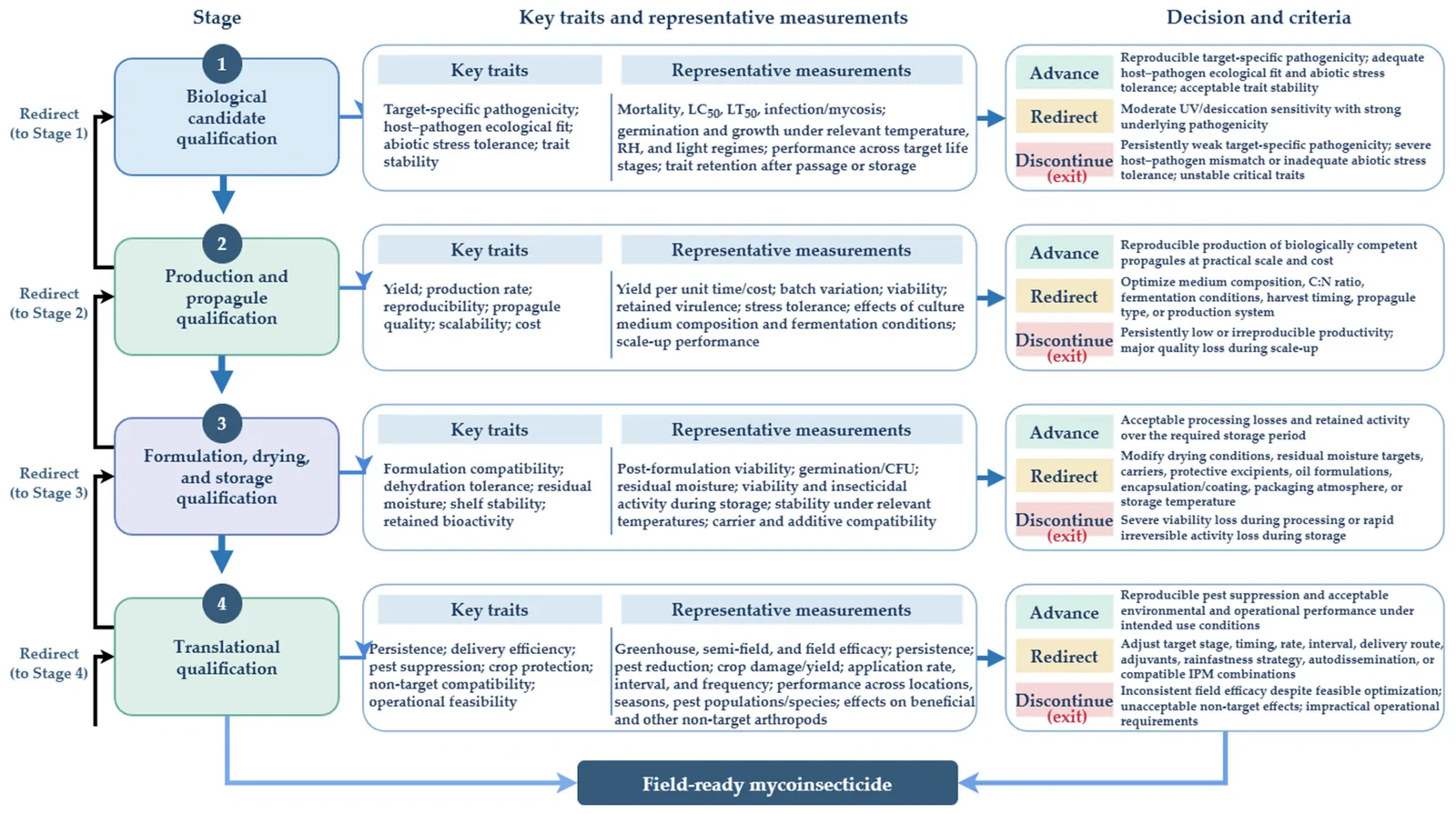
Operational decision framework for advancing entomopathogenic fungi toward mycoinsecticide development. Note: Inclusion of a trait in the framework indicates biological or developmental relevance and does not necessarily imply that it has been validated as an independent predictor of field performance.

The final phase of evaluation should establish whether the formulated product performs reliably under greenhouse, semi-field, and field conditions across relevant locations, seasons, and pest populations or species [76]. Persistence, delivery efficiency, pest suppression, crop protection, compatibility with beneficial organisms and non-target arthropods, together with broader environmental compatibility, and operational feasibility become increasingly important as testing approaches realistic deployment [77]. Progression through development should depend on accumulated evidence across biological, technological, and translational dimensions rather than on a single screening endpoint. Within this framework, criteria for advancement should be defined prospectively according to the intended product profile rather than by universal numerical cutoffs. These criteria should consider the target pest and life stage, deployment environment, production system, required storage period, application method, and, where available, a relevant experimental or commercial benchmark. Critical traits should meet predefined minimum requirements, whereas weaker performance in compensable traits may remain acceptable if production, formulation, or deployment technologies can restore product-level performance without creating prohibitive losses in viability, efficacy, reproducibility, operational feasibility, or cost (Figure 2).

Trait limitations should also be interpreted according to their severity and technological compensability. Consistently poor target-stage pathogenicity, severe host–pathogen mismatch, or major instability may justify discontinuation, whereas moderate UV sensitivity, desiccation sensitivity, or limited persistence may be mitigated through production, formulation, application timing, or delivery technology [78].

An illustrative development scenario shows how this framework can support contingency decisions. A candidate with strong target-stage pathogenicity but inadequate heat, UV, or desiccation tolerance may remain developmentally viable if the limitation can be reduced through nutritional conditioning, protective additives, oil or encapsulated formulations, or a more favorable application window. If viability instead declines substantially during drying or storage, alternative propagule types, drying conditions, residual moisture targets, carriers, protective excipients, packaging systems, or storage temperatures can be evaluated before the candidate is discontinued. When a stable formulated product still performs inconsistently under field conditions, deployment may be redirected through changes in target-stage, application timing, rate, interval, or delivery route. Compatible combination strategies involving other microbial agents, natural enemies, pheromone-based systems, or other IPM components may also be evaluated when a single fungal product provides insufficient control, but their compatibility and potential additive, synergistic, or antagonistic effects should be tested for the specific organisms, formulations, doses, and application sequences involved. Persistent weak pathogenicity, major phenotypic instability, irreproducible production, or unacceptable non-target effects represent stronger reasons for terminating development rather than continuing to invest in technological compensation [9,79].

Trait priorities are likewise application-dependent. Foliar products require greater emphasis on UV tolerance, desiccation tolerance, adhesion, and rain fastness, whereas soil products depend more strongly on persistence, moisture responsiveness, and the capacity to produce infective propagules under soil conditions. Autodissemination systems place greater weight on adhesion, horizontal transmission, and persistence.

Progression from a field-performing candidate to a usable mycoinsecticide also depends on product quality, safety, regulatory readiness, and commercial feasibility. Strain identity should remain traceable to an authenticated reference stock, and product specifications should define appropriate quality control criteria for viable propagule concentration, purity, infectivity, batch consistency, and storage stability [80]. Safety evaluation should address both relevant non-target organisms and human health under realistic exposure scenarios. Human health assessment should consider potential pathogenicity and infectivity, toxicity or toxigenicity, and allergenic or sensitizing effects, particularly through inhalation, dermal contact, or ingestion where these routes are relevant. Occupational exposure of production workers and applicators should also be considered, especially where repeated exposure to viable propagules may occur. Regulatory requirements vary among jurisdictions, but product registration generally requires adequate characterization of the microbial active ingredient and product, together with evidence of manufacturing consistency, efficacy, safety, and stability. Commercial feasibility further depends on reproducible manufacture, shelf life, packaging and distribution, application practicality, and cost at the intended use rate.

The framework should be interpreted as an evidence-informed development structure rather than a validated predictive model. Repeated studies support the developmental relevance of target-specific pathogenicity, host–pathogen ecological fit, abiotic stress tolerance, production conditions, propagule type, formulation, drying, and storage, but direct evidence that individual laboratory or product-level measurements consistently predict field performance remains limited. Some elements of the framework, particularly technological compensability, redirection strategies, and the strain–propagule–process concept, represent integrative hypotheses supported by mechanistic evidence and case studies and require further prospective validation across different EPF systems.

Standardized evaluation is essential throughout this development pathway. Strain identity, passage history, propagule type, production conditions, viable dose, formulation, target-stage, environmental conditions, and application route should be reported consistently. For development and quality-control purposes, strain identity and critical phenotypic traits should also be reverified at defined propagation and production checkpoints against an authenticated reference stock. Even basic quality indicators can vary substantially with methodology; in formulated *B. bassiana* and *M. anisopliae* products, an indirect CFU method markedly underestimated viable conidial concentrations compared with a standardized direct germination method [81]. The next priority is to determine which laboratory and product-level traits are genuinely predictive of field performance, whether candidate rankings remain stable after production and formulation, and how strain–propagule–process and environment interactions shape final efficacy.

## 5. Conclusions

The development of entomopathogenic fungi into effective mycoinsecticides requires evaluation across the full pathway from isolate selection to field deployment. Virulence remains an essential starting point, but laboratory pathogenicity alone provides only a partial indication of development potential. Target-specific pathogenicity, host–pathogen ecological fit, abiotic stress tolerance, and trait stability determine whether an isolate is biologically suitable for advancement, while production fitness, propagule type, formulation compatibility, drying tolerance, and storage stability determine whether that potential can be converted into a practical product.

A key implication is that the phenotype observed during initial screening may differ substantially from the phenotype ultimately delivered in the field. Production conditions and propagule type can alter stress tolerance, virulence, and shelf life, making the strain–propagule–process combination a more informative development unit than strain identity alone. Field performance then emerges from further interactions among the formulated product, target pest, environment, crop, and delivery strategy. Successful translation also requires authenticated strain identity, reproducible product quality, appropriate safety evaluation, regulatory acceptance, and commercial feasibility.

The proposed multi-trait framework integrates these biological, technological, and translational dimensions into a continuous development process. It also distinguishes critical deficiencies that may terminate development from weaknesses that can potentially be mitigated through production, formulation, or application technology. Because trait importance varies among deployment scenarios, candidate evaluation should remain context-dependent rather than rely on a universal weighting system.

Future progress will depend increasingly on identifying traits that reliably predict downstream performance. Linking standardized laboratory measurements with production-scale quality, formulation behavior, and replicated field outcomes should improve candidate selection, reduce development attrition, and support more efficient translation of promising entomopathogenic fungi into reliable mycoinsecticides.

## Figures and Tables

**Table 1:** Comparison of the proposed framework with representative previous approaches.

Approach	Primary Emphasis	Typical Development Perspective	Relationship to the Present Framework
**Virulence-centered and multi-trait isolate screening**	Pathogenicity, virulence, environmental tolerance, and related isolate traits	Identifies biologically promising isolates under defined laboratory conditions	Retained as the biological qualification component, but candidate ranking is subsequently reassessed after production, formulation, and deployment
**Isolation-to-field development approaches**	Sequential progression from isolation and pathogenicity testing toward production and field evaluation	Describes major steps required to move an EPF isolate toward practical use	Extended by explicitly linking each development point to advance, redirect, or discontinue decisions and by distinguishing compensable from development-limiting weaknesses
**Production and formulation-focused approaches**	Fermentation, propagule production, drying, formulation, storage, and product stability	Optimizes manufacturability and protection of fungal propagules	Integrated with upstream candidate qualification and downstream field validation; production is treated as a source of phenotype change rather than only a manufacturing step
**Present multi-trait development framework**	Biological qualification, product qualification, and translational qualification across the isolate-to-field pathway	Treats development as a sequence of context-dependent decisions	Distinguishes strain phenotype from process-shaped product phenotype; uses the strain–propagule–process combination as a product-stage unit; separates compensable from non-compensable limitations; links measurements to advance, redirect, and discontinue decisions

## Data Availability

The original contributions presented in this study are included in the article.
